# A small-molecule ARTS mimetic promotes apoptosis through degradation of both XIAP and Bcl-2

**DOI:** 10.1038/s41419-020-2670-2

**Published:** 2020-06-25

**Authors:** Dana Mamriev, Ruqaia Abbas, Franca-Maria Klingler, Juliana Kagan, Nir Kfir, Alastair Donald, Keren Weidenfeld, David W. Sheppard, Dalit Barkan, Sarit Larisch

**Affiliations:** 1 Cell Death and Cancer Research Laboratory, Department of Human Biology and Medical Sciences, University of Haifa, Haifa, 31905 Israel; 2 The Laboratory of Tumor Dormancy and Metastasis, Department of Human Biology and Medical Sciences, University of Haifa, Haifa, 31905 Israel; 3 BioSolveIT GmbH, Sankt Augustin, Germany; 4 Sheppard R&D Consultancy Ltd, Suffolk, United Kingdom

**Keywords:** Ubiquitin ligases, Ubiquitylation

## Abstract

Many human cancers over-express B cell lymphoma 2 (Bcl-2) or X-linked inhibitor of apoptosis (IAP) proteins to evade cell death. The pro-apoptotic ARTS (Sept4_i2) protein binds directly to both Bcl-2 and XIAP and promotes apoptosis by stimulating their degradation via the ubiquitin-proteasome system (UPS). Here we describe a small molecule, A4, that mimics the function of ARTS. Microscale thermophoresis assays showed that A4 binds XIAP, but not cellular inhibitor of apoptosis protein 1 (cIAP1). A4 binds to a distinct ARTS binding pocket in the XIAP-BIR3 (baculoviral IAP repeat 3) domain. Like ARTS, A4 stimulated poly-ubiquitylation and UPS-mediated degradation of XIAP and Bcl-2, but not cIAP1, resulting in caspase-9 and -3 activation and apoptosis. In addition, over-expression of XIAP rescued HeLa cells from A4-induced apoptosis, consistent with the idea that A4 kills by antagonizing XIAP. On the other hand, treatment with the SMAC-mimetic Birinapant induced secretion of tumour necrosis factor-α (TNFα) and killed ~50% of SKOV-3 cells, and addition of A4 to Birinapant-treated cells significantly reduced secretion of TNFα and blocked Birinapant-induced apoptosis. This suggests that A4 acts by specifically targeting XIAP. The effect of A4 was selective as peripheral blood mononuclear cells and normal human breast epithelial cells were unaffected. Furthermore, proteome analysis revealed that cancer cell lines with high levels of XIAP were particularly sensitive to the killing effect of A4. These results provide proof of concept that the ARTS binding site in XIAP is “druggable”. A4 represents a novel class of dual-targeting compounds stimulating apoptosis by UPS-mediated degradation of important anti-apoptotic oncogenes.

## Introduction

Apoptosis is essential to maintain tissue homeostasis and protect against various diseases, including cancer^1^. Caspases, a family of cysteine proteases, are the central executioners of apoptosis^2^. The activity of caspases is inhibited through the action of the inhibitor of apoptosis (IAP) proteins^3^. There are eight human IAPs, yet only X-linked IAP (XIAP) directly inhibits caspases, whereas cellular inhibitor of apoptosis proteins (cIAPs) prevent the formation of pro-apoptotic signalling complexes at the extrinsic apoptotic pathways. XIAP contains three baculoviral IAP repeats (BIRs), which serve as protein–protein interaction domains for direct binding and inhibition of caspases 3, 7 and 9^4,5^. In addition, it contains an ubiquitin-associated domain, which enables the binding of poly-ubiquitin conjugates and a RING domain required for its E3 ligase activity^6–8^. The regulation of apoptosis critically relies on regulated protein degradation by the ubiquitin-proteasome system^9–11^. XIAP functions as an E3 ligase to promote degradation of several pro-apoptotic proteins, such as SMAC and ARTS^12–14^.

Apoptosis is also regulated by B cell lymphoma 2 (Bcl-2) family members, which control mitochondrial outer membrane permeabilization (MOMP)^15,16^. This family contains pro- and anti-apoptotic proteins that can form complexes via a common BH3 domain^15,17–20^. Importantly, high levels of the anti-apoptotic Bcl-2 protein are characteristic of many haematologic malignancies as well as certain solid cancers^15,21–24^.

To stimulate apoptosis, the function of IAPs needs to be overcome. This occurs by the action of IAP antagonists, such as SMAC/Diablo^25,26^, Omi/HtrA2^27^ and ARTS^28^. ARTS (Sept4_i2) is a pro-apoptotic protein encoded by the Septin 4 (*Sept4*) gene and the only splice variant of *Sept4* that promotes apoptosis^29,30^. Studies in human and mice show that ARTS acts as a tumour suppressor protein. *Sept4/ARTS*-deficient mice develop various spontaneous tumours, and they have highly accelerated lymphomagenesis in the Eu-Myc model^31,32^. In addition, ARTS expression is lost in >70% of leukaemia patients, in 50% of lymphoma patients and in a significant fraction of hepatocellular carcinoma patients^31,33^ (S. Larisch and H. Steller, unpublished data). In living cells, ARTS localizes to the mitochondrial outer membrane (MOM) where it binds to Bcl-2^13^. Upon induction of apoptosis, ARTS translocates to the cytosol and directly binds and antagonizes XIAP. This leads to de-repression and release of non-lethal active caspases from XIAP upstream of MOMP. In turn, these caspases can cleave substrates, such as Bid, resulting in MOMP and execution of apoptosis^13,34^. ARTS stimulates the degradation of XIAP by promoting its ubiquitylation^13,35^. In addition, ARTS acts as a scaffold to bring XIAP into close proximity with Bcl-2 and promote its degradation^34^. Thus, ARTS functions as a dual antagonist of both XIAP and Bcl-2 upstream of MOMP. Importantly, ARTS acts as a physiological antagonist of XIAP. *Sept4/ARTS*-deficient mice express elevated levels of XIAP protein, demonstrating that ARTS limits the levels of XIAP in vivo. Moreover, knockdown of ARTS in HeLa cells and intestinal epithelial cells from Sept4/ARTS knockout (KO) mice are protected against apoptotic cell death^13,36^. In addition, *Sept4/ARTS*-deficient mice have increased numbers of hematopoietic stem and progenitor cells (HSPCs) and hair follicle stem cells that are resistant to apoptosis^31,37^. Furthermore, the resistance of *Sept4/ARTS*-null HSPCs to apoptosis and the cell-autonomous lymphoproliferation is suppressed by the loss of XIAP function in *Sept4/ARTS/XIAP* double-KO mice^31^. Collectively, these results demonstrate the important physiological role of ARTS in regulating apoptosis and as a tumour suppresor in vivo through its role as a specific XIAP antagonist.

ARTS differs from all other known IAP antagonists by its distinct mode of binding to XIAP^14,38^. Moreover, ARTS specifically induces degradation of XIAP and Bcl-2^13,28,34^. Significantly, over-expression of both XIAP and Bcl-2 contributes to tumorigenesis and have become major targets for developing anti-cancer therapeutics^39–42^. IAP antagonists were initially designed based on the N-terminal peptide sequence AVPI found in the *Drosophila* Reaper/Hid and SMAC/Diablo^5,43,44^. SMAC mimetics (SMs) bind with high affinity to cIAPs and lower affinity to XIAP and they can degrade cIAPs, but not XIAP^38,45–48^. Here we describe the identification of the first ARTS-mimetic small molecule, A4. This compound binds directly to the unique binding site of ARTS in XIAP-BIR3, but not to cIAP1. A4 promotes proteasome-mediated degradation of both XIAP and Bcl-2, caspase activation and apoptosis. Over-expression of XIAP inhibits A4-induced cell death, consistent with the idea that XIAP is a major target for A4.

## Materials and methods

### Cell line culture and reagents

HeLa (human cervical cancer cells), A375 (human malignant melanoma cells), Jurkat (human leukaemia T cells) and HEK-293-T (human embryonic kidney cells) were purchased from ATCC.

The DKO BAK/BAX MEFs (mouse embryonic fibroblasts) were kindly provided to us by Dr. Joe Opferman, St. Jude, Memphis, TN, USA, and by Dr. Reuven Stein, Tel-Aviv University, Israel.

MEFs cells, HeLa, A375 and HEK-293-T cells were grown in complete DMEM medium (1% sodium pyruvate, 1% l-glutamate, 1% Pen-strep and 10% fetal bovine/calf serum).

Jurkat and T47D (human metastatic ductal breast carcinoma cells) cells were grown in complete RPMI medium (1% sodium pyruvate, 1% l-glutamate, 1% Pen-strep and 10% heat-inactivated fetal bovine/calf serum).

184A1 (normal human breast epithelial cells) were grown in DMEM/F12 complete medium (1% sodium pyruvate, 1% l-glutamate, 1% Pen-strep, 5% donor horse serum, 100 ng/ml cholera toxin, 20 ng/ml epidermal growth factor, 0.5 mg/ml hydrocortisone, 10 µg/ml insulin).

All cell lines were checked for mycoplasma and kept under passage 10.

Staurosporine (STS) was purchased from Fermentek (cat#62996-74-1.5) and Birinapant from Biovision (cat#5297).

### Preparation of A4 stock and work solution

The A4 small molecule (MW 440.92 g/mol as powder, SMILES: COC(=O)c1[nH]c2ccc(Cl)cc2c1NC(=O)C[NH + ]1CC[NH + ](Cc2ccccc2)CC1) was purchased from eMolecules, Inc., eMolecule ID: 4424446 (Supplier InterBioScreen STOCK2S-13772). A4 was dissolved in dimethyl sulfoxide (DMSO) to a stock solution of 30–50 mM, followed by intensive pipetting and centrifugation at 300 × *g* for 30 s. Next, the A4 suspension was incubated in a 37 °C bath for 1 min, mixed thoroughly by pipetting and spun down again. A4 stock solution was aliquoted in Eppendorf tubes (7–10 µl/tube) and stored in −80 °C. Aliquots were only used once to avoid freeze and thaw of the compound. Before using in an experiment, an A4 aliquot was thawed, spun down (same settings) and mixed by tapping gently at the lower part of the Eppendorf tube. Next, the compound solution was diluted 1:100 in warm complete medium in a 15 ml conical tube to a concentration of 0.3–0.5 mM and mixed well by tilting the closed vial up and down (do not vortex). The diluted A4 solution was then diluted again to the desired final concentration (10–30 µM) and added to the cells.

### Antibodies

ARTS—mouse monoclonal anti-ARTS antibody, specifically targeting the unique c-terminal sequence of ARTS (but not other Septin 4 splice variants) at a dilution of 1:1000 (Sigma A4471).

XIAP—mouse monoclonal anti-XIAP antibody (BD cat#610717) at a dilution of 1:1000. Or rabbit polyclonal anti-XIAP (sc-11426) at a dilution of 1:2000.

Bcl-2—mouse monoclonal anti-Bcl-2 antibody (BD cat#610538) at a dilution of 1:1000. Or rabbit polyclonal anti-Bcl-2 by Proteintech (26593-1-AP) or DB Biotech (P22-A cat#DB 132-0.05) at a dilution of 1:5000 for both.

Actin—mouse monoclonal anti-actin antibody (ImmunoTM cat#08691002) at a dilution of 1:50,000.

Cleaved PARP (cPARP)—rabbit polyclonal anti-PARP antibody (Abcam cat#ab4830) at a dilution of 1:1000.

Ubiquitin—mouse monoclonal anti-ubiquitin (Santa Cruz sc-8017) at a dilution of 1:2000.

Myc—monoclonal mouse anti-Myc (Cell Signaling cat#2276s) at a dilution of 1:1000.

Tubulin—monoclonal rat anti-tubulin (Abcam cat#YOL1/34) at a dilution of 1:6000.

GAPDH—polyclonal goat anti-GAPDH (Abcam cat#ab9483) at a dilution of 1:2000.

BAX—rabbit polyclonal anti-Bax (Proteintech 50599-2-Ig) at a dilution of 1:6000.

cIAP1—goat polyclonal anti-cIAP1/HIAP-2 antibody (R&D cat#AF8181) at a dilution of 1:1000.

Cleaved caspase-3—for immunofluorescence we used purified rabbit anti-active caspase-3 antibody clone C92-605 (BD cat#559565) at a dilution of 1:400.

Cleaved caspase-3—to detect cleaved caspase-3 by western blot we used rabbit monoclonal antibody anti-active Caspase-3 Asp175 (BD cat#cs9664) at a dilution of 1:1000.

### Screening of A4 killing effect on 94 tumour cell lines

Screening of 94 different cancer cell lines spanning over 18 human tissues was performed by Oncolead Ltd. The company used an identical platform to the one NIH, NCI-60 uses to assess the effect of a compound on the viability of a variety of tumour cell lines. Briefly, cells are treated with a series of six dilutions of a compound for 72 h (compound is replenished daily). After 72 h, cells were fixed (living cells are attached to the plate), and then washed to remove dead and dying cells. Cells were then stained with a dye (sulforhodamine B) that dyes all proteins purple, which is measured by a plate reader and provides quantification of living cells. The number of cells treated with the compound is compared to the number of cells treated with DMSO (solvent). The inhibitory concentration (IC) value was calculated by taking the number of cells after 72 h of treatment and dividing by the number of cells after 72 h treatment with DMSO (C). IC50 is the value calculated from the dose–response plot, which is the concentration of the compound that has 50% decrease of the IC.

#### Cell viability assays

XTT Cell Proliferation Kit (Biological Industries cat#20-300-1000) and Presto blue cell viability reagent (Invitrogen cat#A13262) were used according to the manufacturer’s instructions. Six thousand to 10,000 cells per well were seeded in Falcon® 96-well Clear Flat Bottom TC-treated Culture Microplate (#353072) and grown for 24 h. Treatment with A4 was added for 24 h following incubation at 37 °C with XTT or the Presto blue reagents for 2 h. The intensity of the colour was measured using BioTeK ELISA synergyHT microplate reader (for XTT 450 nm excitation and 630 nm emission, for Presto blue assay 560 nm excitation and 590 nm emission). Using both these viability assays, we determined cell viability by normalizing the results in the treated cells to cells treated with DMSO (fold change to DMSO). Dot plots are composed of independent experiments, and each independent experiment was done in triplicates or more.

### Caspase-9 activity assay

One million cells per well were seeded in 6-well plates. After 24 h, the cells were treated with 20 μM A4 for 3, 6 and 24 h. The cells were collected with phosphate-buffered saline (PBS) and washed again with PBS. The cells were centrifuged at 500 × *g* for 10 min. Cleaved caspase-9 activity was then measured in these samples by Caspase-9 Fluorometric Assay Kit (by BioVision cat#K118) according to the manufacture’s protocol. Fluorescence levels were measured by an ELISA spectrophotometer (400 nm excitation and 505 nm emission). The results were normalized by the absolute value (OD) of the DMSO control to evaluate the caspase-9 activity.

#### Caspase-Glo 3/7 assay system (Promega cat#G8090)

Cells were cultured in 96 wells (8000 cells/well) and were treated with A4 (20 µM). After overnight incubation at 37 °C, 5% CO_2_ incubator Caspase-Glo® reagent (Promega, USA) was added to each well according to the manufacturer’s instructions and plates were incubated at room temperature for 1 h. Luminescence of each sample was measured using a plate-reading infinite M200PRO, TECAN luminometer.

### Annexin V/PI staining

Jurkat cells were treated with A4 plus or minus the caspase inhibitor Q-VD-Oph (ab141421) for 24 h (1–10 µM) and stained for Annexin V/PI according to the manufacturer’s instructions (Novus cat#NBP2-29373). Q-VD-Oph was added 30 min prior to the addition of A4. Flow cytometry was performed using FACSCanto II (BD) and data were analysed using the FlowJo software (Treestar).

### Immunofluorescence detecting active caspase-3

For immunofluorescence and confocal microscopy, cells were grown on cover glasses (Marienfeld, Lauda-Konigshofen, Germany) and treated with A4 for either 6 or 24 h. Next, the cells were washed three times with PBS and fixed with 4% paraformaldehyde for 15 min at room temperature (RT), followed by three additional PBS washes. Permeabilization was performed with 0.1% Triton X-100 in 50 mM Tris pH 7.2 for 3 min, after which the cells were washed with PBS and incubated in blocking buffer (20% normal goat serum and 1% BSA in PBS) for 30 min at RT. Cells were then incubated with the antibody for cleaved caspase-3 (1:400 by BD cat#559565) diluted in PBS containing 1% BSA, for 1 h at RT, followed by three PBS washes. The cover glasses were then incubated with the secondary antibody for 1 h at RT. After washing with PBS, they were mounted using VECTASHIELD mounting medium with 4′, 6-diamidino-2-phenylindole. Immunofluorescent images were taken with a Nikon A1R confocal microscope.

### Treatment with proteasome inhibitors

Cells were pre-incubated with 20 µM of MG-132 (APExBIO cat#A2585) or Bortezomib (APExBIO cat#A2614) for 6 h. Twenty micromoles of A4 was added in the last 2 h of incubation with the proteasome inhibitors.

### Site-directed mutagenesis of XIAP expression construct

We have mutated two key amino acids at the distinct binding site of ARTS within XIAP-BIR3. Using the GFP-WT-XIAP expression construct, we mutated amino acids S278A and N280A (GFP-DM-XIAP). As a control we used the GFP-WT-XIAP and the GFP-empty-vector. A detailed description is in the Supplementary Materials and methods.

### Transfections reagents

For transient transfections, the following reagents were used according to the manufacturer’s instructions: Transit-X2 (Mirus), jetPEI (Polyplus) and PolyJet (SignaGen).

### SDS-PAGE and western blot analysis

Cells were lysed in whole-cell extract buffer [25 mM HEPES, pH 7.7, 0.3 M NaCl, 1.5 mM MgCl_2_, 0.2 mM EDTA, 0.1% Triton X-100, 100 μg/ml phenylmethylsulfonyl fluoride (PMSF) and protease inhibitor cocktail (Roche, 1:100 dilution)] and placed on ice for 30 min (vortexing once after 15 min). After 30 min, the samples were centrifuged at 13,000 × *g* for 10 min at 4 °C. The supernatants containing total protein were measured for protein concentration using Bio-Rad Protein Assay Dye Reagent Concentrate Kit. Forty to 100 µg protein was separated by sodium dodecyl sulfate-polyacrylamide gel electrophoresis (SDS-PAGE) (12%), followed by transfer to a PVDF membrane. The membranes were blocked with 5% (w/v) non-fat dried skimmed milk powder in PBS supplemented with 0.05% Tween-20 (PBS-T) for 1 h at RT. Next, primary antibodies were added at 4 °C overnight or for 2 h at room temperature. Membranes were then incubated with secondary antibody for 1 h at RT and washed three times for 15 min each with PBS-T. Western Bright ECL (Advansta) was added to the membrane for 30–60 s and analysed using Image Quant LAS-4000 (GE Healthcare Life Sciences) and Image Quant LAS-4000 software (GE Healthcare Life Sciences).

### Co-immunoprecipitation

HEK-293-T cells were transfected with either GFP-empty-vector, GFP-WT-XIAP or GFP-DM-XIAP and allowed them to express the constructs for 24 h. All samples were co-transfected with 6-Myc-ARTS (Myc tag is located at the N′ terminus). Next, the cells were harvested and lysed with radioimmunoprecipitation assay (RIPA) buffer (Tris-HCl pH 7.5 50 mM, NaCl 150 mM, NP-40 (Igepal) 0.3%) containing protease inhibitor cocktail (Complete, Roche) and 100 μg/ml PMSF. Antibodies were used at 5 µg per 1000 µg protein and were incubated overnight rotating at 4 °C. The next day, agarose beads conjugated to protein A/G (Santa Cruz Biotechnology) were added for 4 h with rotation at 4 °C. Samples were centrifuged at 4 °C for 5 min and washed five times with RIPA buffer. Proteins were eluted from beads by 10 min of boiling in sample buffer and separated on 12% SDS-PAGE gel, followed by western blot analysis.

### In vivo ubiquitylation assay

All cells were transiently transfected with a Ub-HA (ubiquitin-tagged with HA) construct and treated with proteasome inhibitor (Bortezomib or MG-132, at 20 µM for 6 h). In addition, for Bcl-2 in vivo ubiquitylation assay, cells were co-transfected with Ub-HA and GFP-vector/Bcl-2-GFP (GFP tag is attached to the N′ terminus of Bcl-2). After 4 h of proteasome inhibitor incubation, A4 (20 μM) or DMSO was added to the medium for an additional 2 h. After 6 h of Bortezomib/MG-132 along with 2 h of A4 treatments, the cells were harvested and lysed using RIPA buffer (Tris-HCl pH 7.5 50 mM, NaCl 150 mM, NP-40 (Igepal) 0.3%) containing protease inhibitor cocktail (Complete, Roche), 100 μg/ml PMSF, 5 mM *N*-ethylmaleimide and 5 mM iodoacetamide to preserve ubiquitin chains. Following 15 min of centrifugation (10,000 × *g*, 4 °C), the supernatant was transferred into a clean Eppendorf tube. A ubiquitination assay using immunoprecipitation with anti-XIAP antibody (sc-11426 or BD #610717) (endogenous XIAP was pulled down) or anti-Bcl-2 (Proteintech #26593-1-AP or DB Biotech P22-A cat#DB 132-0.05) (over-expressed Bcl-2 was pulled down) was performed as described above with 5 µg antibody per 1000 µg protein. Poly-ubiquitylated forms of Bcl-2 or XIAP were detected using anti-Ub Ab (Santa Cruz sc-8017).

### SKOV-3 rescue assay and evaluation of Birinapant-induced apoptosis

The SKOV-3 rescue assay was performed based on Gaither et al.^49^. Specifically, SKOV-3 cells were seeded in 96-well plates in replicates of four for each treatment. After 24 h, the cells were treated with Birinapant (1 µM) for 24 h in the presence or absence of A4 (2.5 and 5 μM). The cells were then washed once with 100 μl of PBS. Next, 100 μl of Presto blue reagent (diluted 1:5 in PBS) was added to each well and the cells were incubated at 37 °C for 2 h. After the 2-h incubation, absorbance was measured using a fluorescence spectrometer (emission 560 nm, excitation 590 nm).

### Detection of TNF-α levels in condition media

SKOV-3 cells were treated for 24 h with A4 alone (10 µM), Birinapant alone (1 µM) and a combination of the two compounds. DMSO was used as a negative control. Tumour necrosis factor-α (TNFα) levels in supernatants were measured using ELISA Max standard sets (Cat#430203 BioLegend), according to the manufacturer’s protocol.

### Computational screen

Three hundred thousand commercially available molecules were selected from a set of ~3 million and were screened using LeadIT and SeeSAR software suits from BioSolveIT. This computational screen-identified compounds with predicted binding affinities in the micromolar to nanomolar range, as assessed by the HYDE scoring function^50^. The 100 top-ranked molecules exhibiting best docking scores were determined. The ARTS unique binding site in XIAP-BIR3 was extrapolated by analysing XIAP-SMAC crystal structures from the PDB and our data, described by Bornstein et al.^14^. A detailed description is provided in the Supplementary Materials and methods.

### Computational analysis of ARTS versus SMAC binding sites in XIAP-BIR3

Using the site mapping information on the unique ARTS binding site in XIAP-BIR3^14^, we performed a preliminary virtual screen using Glide^51^ into the 3HL5.PDB crystal structure^52^. Compounds identified as described above were docked into the model both with and without water present in the active site using a standard protocol.

### MST binding assays

Microscale thermophoresis (MST) binding assays were performed by CreLux, a WuXi AppTech company in Germany, using recombinant ARTS, XIAP, Bcl-2 and cIAP1 proteins. Specifically, for performing experiments with untagged XIAP, a fluorescent label (NT650) was covalently attached to the protein (Maleimide coupling). The labelling was performed in a buffer containing 50 mM HEPES pH 7.0, 150 mM NaCl and 0.005% Tween-20. A detailed description is provided in the Supplementary Materials and methods.

### Proteomic data

All proteomic data for cell lines available at EMBL-EBI Expression Atlas repository was downloaded (https://www.ebi.ac.uk/gxa/home). This included in total 13 datasets, of which 10 included cell lines represented in our set of 93 cell lines. Proteomic data was retrieved for 40 out of 93 cell lines. From each dataset, data for four genes were curated: ACTB, GAPDH, XIAP and Bcl-2. The first two were used for normalization of expression levels of XIAP and Bcl-2. All data were log 10 transformed and ratios between XIAP or Bcl-2 and either of the normalizing genes were calculated. A Wilcoxon’s rank sum test was used to compare the IC50 values (μM) between the different types of tumour cells. The test was performed for several pairs of cancer types (Supplementary Table 1).

### Data filtering and correlation analysis

In order to inspect relatedness between A4 IC50 and XIAP or Bcl-2 expression, the cell lines were catalogued based on their tissue/organ of origin, and analysis was performed on tissues/organs represented by at least four different cell lines (Supplementary Tables 1 and 2). We further excluded values (expression ratios) that were >1.0, considering such values for these specified genes are likely biased. Indeed, in these samples expression levels of GAPDH or Actin were 2–3 orders of magnitude lower than those found in other samples. We then calculated correlation between XIAP expression ratios using both Pearson and Spearman correlations. Correlations were calculated and tested for significance using function cor.test in R package “stats”. Correlation was considered significant for *p* < 0.05.

### Statistical analysis

All graphs were made using the PRISM software. Significance was evaluated using PRISM’s one-way analysis of variance. *p* < 0.05 was considered significant.

## Results

### Structure-based computational screen for molecules targeting the unique binding site of ARTS in XIAP-BIR3

**Fig. 1:**
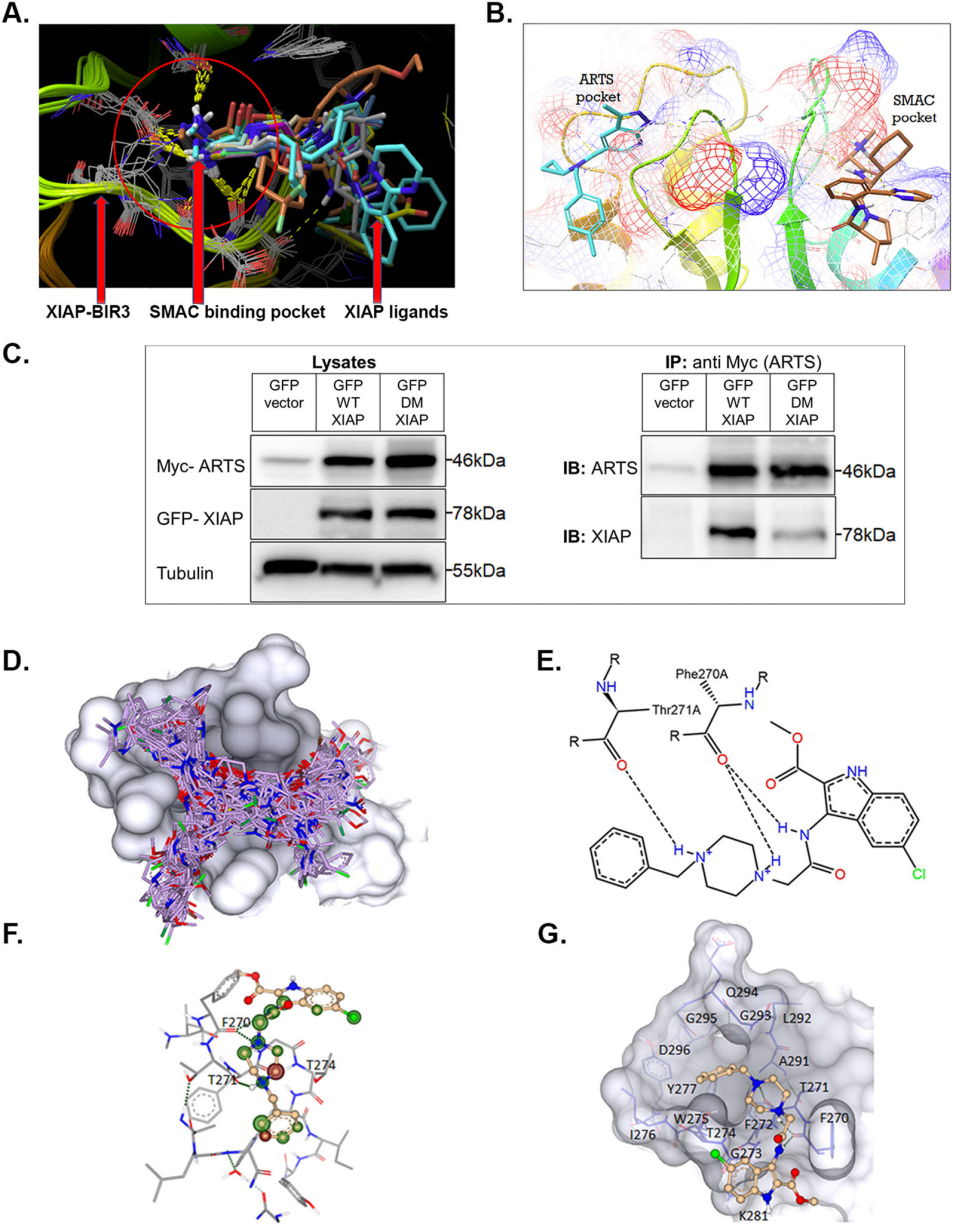
Identification of A4 in a structure-based computational screen for small molecules docking to the unique ARTS binding site within the XIAP-BIR3 domain. **a**, **b** The binding site of ARTS within XIAP-BIR3 is distinct from that of SMAC/Diablo. **a** All previously reported that XIAP ligands bind to the same well-characterized “SMAC domain” in XIAP-BIR3 (“SMAC binding pocket”). Overlay of publicly available crystal structures of the BIR3 domain predicted that all previously described ligands bind exclusively to the SMAC domain. **b** The ARTS and SMAC/Diablo binding pockets within XIAP-BIR3 do not overlap. Schematic representation of the ARTS and SMAC binding pockets. On the left, XIAP-BIR3 is depicted with a virtual compound (in cyan) to illustrate the shape of the pocket. The ARTS binding site is close to the SMAC binding site, but there is no overlap between the two sites. **c** HEK-293-T cells were co-transfected with either 6-Myc-ARTS and GFP-WT-XIAP or 6-Myc-ARTS with GFP-DM-XIAP-S278A-N280A. ARTS was pulled down using anti-Myc antibody followed by immunoblotting with anti-XIAP antibody. Results show reduction in the binding of GFP-DM-XIAP-S278A-N280A to 6-Myc-ARTS compared to binding of 6-Myc-ARTS to GFP-WT-XIAP (*n* = 3). **d**–**g** A structure-based computational screen-identified compound A4 as a strong candidate to bind the ARTS pocket in XIAP-BIR3. **d** The surface of the ARTS binding site in the BIR3 domain of XIAP (in grey) is modelled with the predicted best fit binding positions of the top 100 ligands (in purple, BioSolveIT Ltd). **e**, **f** Interaction of A4 with XIAP-BIR3. **e** Interaction of A4 with XIAP-BIR3 in 2D. The hydrogen bonds between A4 and the backbone carbonyls are depicted as dashed lines, hydrophobic contacts are indicated by the green lines and the interacting amino acids are labelled in green. **f** Binding mode of compound A4 (in bold-beige) to the ARTS binding site. The spheres around each atom of A4 depict the individual contribution to the binding affinity: green indicates positive and red negative contribution, and no sphere means no contribution to the binding. Hydrogen bonds are depicted as dashed green lines. The model predicts that A4 interacts with the backbone of T271, but an alternative possibility is that A4 interacts with the backbone of T274, instead of T271. **g** A4 is displayed in a balls-and-sticks model. The surface of the distinct ARTS binding site in XIAP-BIR3 is shown in grey, and the specific amino acids are represented as blue sticks behind it. A4 is expected to form three H-bond interactions with the protein backbone (dashed green lines) between T271 and a nitrogen of the piperazine ring between F270, and the other nitrogen plus the amide nitrogen.

Both SMAC and ARTS bind to the BIR3 domain in XIAP^14,38,53^. Using the site mapping information of both ARTS and SMAC, we performed an in silico screen using Glide^54^ Schrodinger^55^ into the 3HL5.PDB crystal structure^52^. All XIAP ligands found in the computational screen were bound to the SMAC site (“SMAC binding pocket”, Fig. 1a), but not to the ARTS binding site (Fig. 1b). Figure 1b illustrates the distinct nature of the binding pockets for ARTS and SMAC within XIAP-BIR3. To further demonstrate that the BIR3 domain in XIAP contains a distinct binding site for ARTS, we generated a double mutant expression construct of XIAP (DM XIAP) in which two amino acids were replaced with Alanine (S278A and N280A). Immunoprecipitation assays revealed a strong reduction in the ability of ARTS to bind to this mutant XIAP, as compared to wild type (WT) (Fig. 1c). This confirms that XIAP contains a distinct binding site for ARTS in its BIR3 domain.

Previous efforts have exclusively focused on compounds binding to the SMAC pocket^53,56,57^. To identify compounds that bind specifically to the ARTS binding pocket, a structure-based computational docking screen was performed. Overlay of the 100 top-ranked docking compounds revealed that they cover all the different sub-pockets within the surface of the ARTS-specific binding site (Fig. 1d). This indicates that the binding surface of XIAP-BIR3 was thoroughly explored by the screening protocol. Compound A4, one of the top-ranked molecules, is predicted to form three H-bond interactions with the protein backbone of the distinct ARTS binding site: between T271 and a nitrogen of the piperazine ring and between F270 and the other nitrogen plus the amide nitrogen (Fig. 1e). However, it is also possible that A4 interacts with the backbone of T274 instead of T271 (Fig. 1f, g). Interestingly, the docking map reveals unusually curved docking positions, so that the ligand turns away from the SMAC binding site. This exposes the indole ring to the surface, suggesting that it may be the ARTS-accessible surface (Fig. 1g). A4 is a methyl 5-chloro-3-[[2-[4-(phenylmethyl) piperazin-1-yl]acetyl]amino]-1*H*-indole-2-carboxylat. It conforms well with Lipininski’s rule of five and hence has promising chemical properties for potential future drug development.

### A4 induces caspase-mediated cell death

**Fig. 2:**
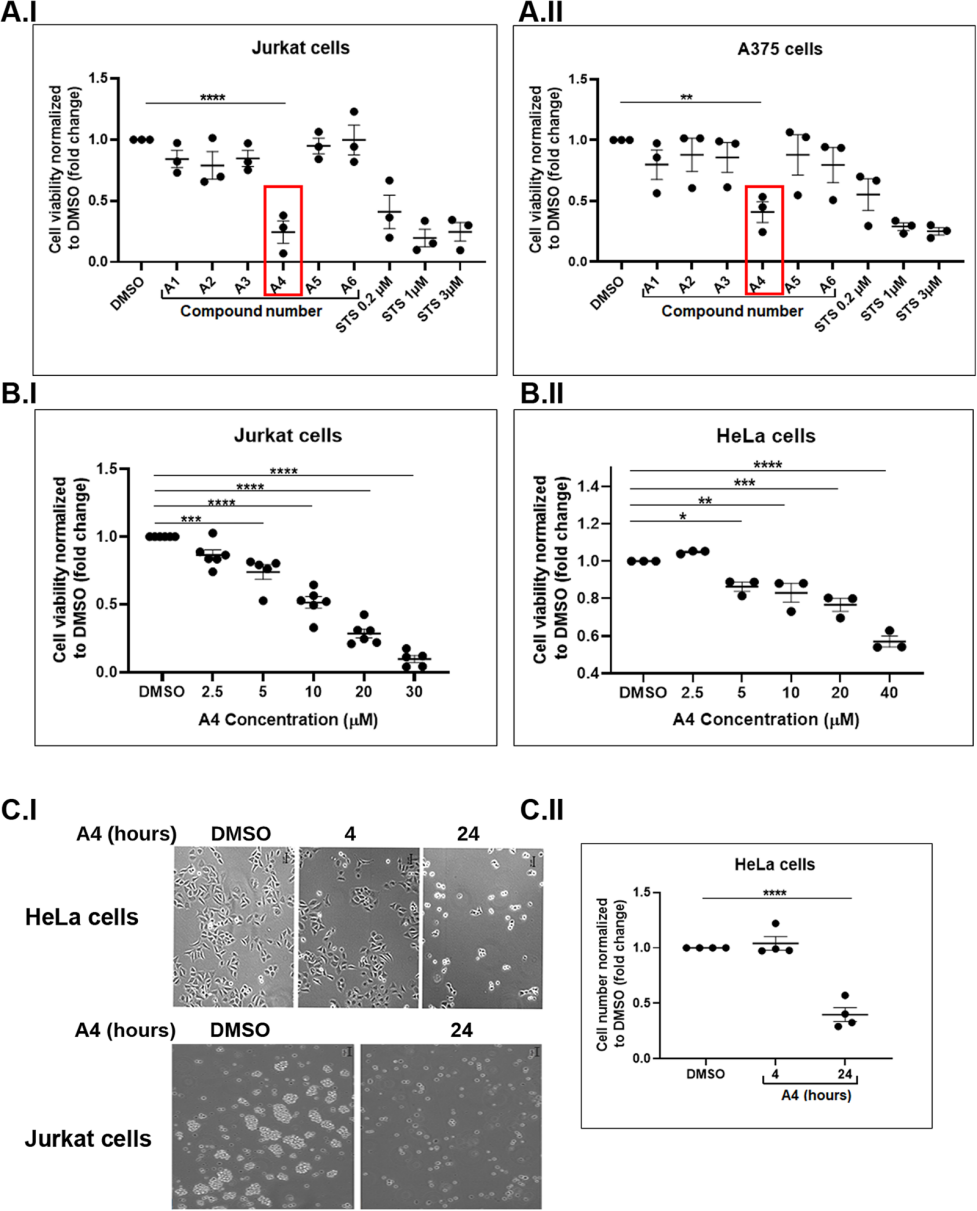
The ARTS-mimetic A4 is a potent inducer of cell death. **a** Dot plots representing cell viability of Jurkat (I) and A375 cells (II) treated for 24 h with different compounds identified in the computational screen. Induction of apoptosis with various concentrations of STS (for 3 h) served as a positive control (*n* = 3). **b** Dose response of A4 (24 h) on cell viability of Jurkat (*n* = 5; I) and HeLa cells (*n* = 3; II). Dots represent an average of triplicates for each independent experiment; bars represent the mean. Error bars; SEM, one-way ANOVA, *p < 0.05, **p < 0.01, ***p < 0.001, ****p < 0.0001. **c** Representative bright-field microscopy images of HeLa and Jurkat cells undergoing apoptosis induced by A4 treatment for 4 and 24 h in HeLa cells and 24 h in Jurkat cells (I). Magnification ×10, scale bar = 50 µm. (II) Quantification of HeLa cell numbers (in I). The average of total cell numbers from five different images (frames) was counted and normalized to DMSO-treated cells (*n* = 4). Dots represent an average of five frames from each independent experiment; bars; SEM, one-way ANOVA, *p < 0.05, **p < 0.01, ***p < 0.001, ****p < 0.0001.

**Fig. 3:**
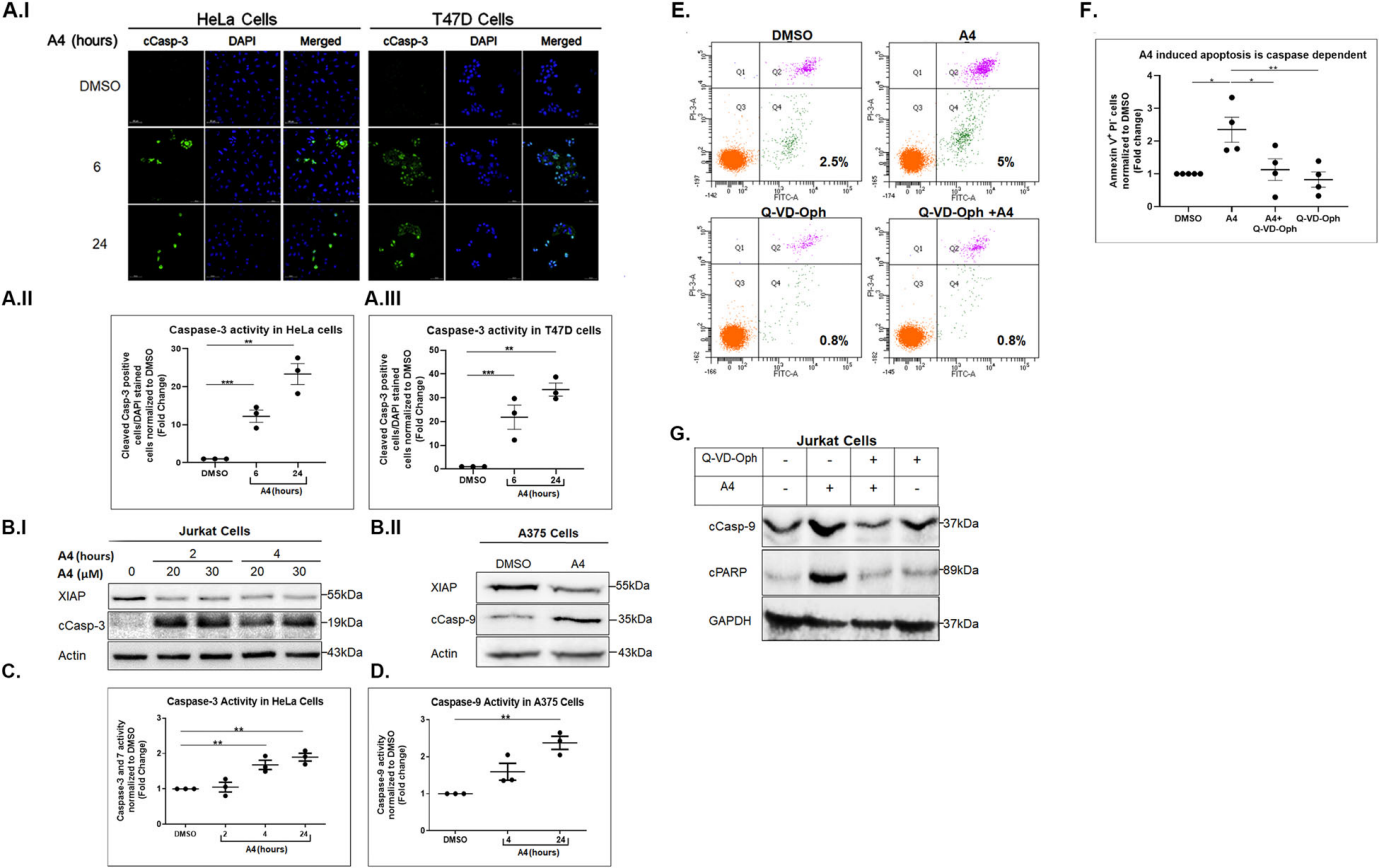
The ARTS-mimetic A4 is a potent inducer of caspase-9- and caspase-3-mediated apoptotic cell death. **a** (I) Immunofluorescence images of cleaved caspase-3 (cCasp-3, green) after 6 and 24 h of treatment with A4. Magnification ×40, scale bar = 50 µm. (II, III) Quantification of the percentage of cells stained positive for activated caspase-3 (in I) normalized to DMSO-treated cells (*n* = 3). Dots represent an average of five frames from each independent experiment; bars; SEM, one-way ANOVA, **p < 0.01, ***p < 0.001. **b** (I, II) Representative Western blot (WB) analysis of XIAP and of cleaved caspase-3 (cCasp-3) (I) or cleaved caspase-9 (cCasp-9) expression in Jurkat cells (I) or in A375 cells (II) upon treatment with A4 (*n* = 3). **c** Caspase-3/7 activity in HeLa cells treated with A4. Data were normalized to DMSO-treated cells (*n* = 3). **d** cCasp-9 activity measured in A375 cells treated with A4 (*n* = 3). Dots represent an average of triplicates for each independent experiment; bars; SEM, one-way ANOVA, **p < 0.01. **e**–**g** Jurkat cells treated with A4 in the presence or absence of Q-VD-Oph. **e** Representative dot plots from FACS analysis of Jurkat cells stained with Annexin V/PI. **f** Quantification of Jurkat cells that were positive for Annexin V staining and negative for PI staining upon treatment with A4 in the absence or presence of Q-VD-Oph (ON) (*n* = 4). Dots represent an average of triplicate experiments. Bars; SEM, one-way ANOVA, *p < 0.05, **p < 0.01. **g** Representative WB for the expression of cCasp-9 and cleaved PARP (cPARP) in Jurkat cells treated with A4 in the absence or presence of Q-VD-Oph (*n* = 3).

The effects of our top-ranked molecules were first tested on A375, a melanoma cell line expressing high levels of XIAP, and a Jurkat T-lymphoma cell line expressing high levels of Bcl-2. This initial functional screen tested for the death-inducing effect of 76 out of the 100 top scoring compounds using the PrestoBlue™ Cell Viability Reagent and XTT Cell Viability Kit. Figure 2a shows representative results for some of these compounds in comparison to the pro-apoptotic reagent STS. From these screens, we identified A4 as a potent cell killer (Fig. 2aI, II and Supplementary Fig. 1a, b). A4 also induced cell death of HeLa, Jurkat and T47D human cancer cells (Fig. 2bII, 2cI, II, 2bI, 2cI and 5cII, respectively). Notably, treating these cells with A4 resulted in increased levels of cleaved caspase-3 and elevated activity of cleaved caspase-3 and -7 (Fig. 3aI–III, 3bI and Fig. 3c, respectively). Moreover, increased levels and activity of cleaved caspase-9 were detected in A375 cells upon treatment with A4 (Fig. 3bII and 3d, respectively), and this was associated with a strong decrease in cell numbers (Supplementary Fig. 1c, d). Finally, using Annexin V/PI FACS analysis and cPARP as an apoptotic marker, we found that treatment with the caspase inhibitor Q-VD-Oph blocked A4-induced apoptosis (Fig. 3e–g). These results indicate that A4, like ARTS, promotes apoptosis through activation of both caspase-3 and -9.

### A4 binds directly to XIAP and stimulates proteasome-mediated degradation of both XIAP and Bcl-2

**Fig. 4:**
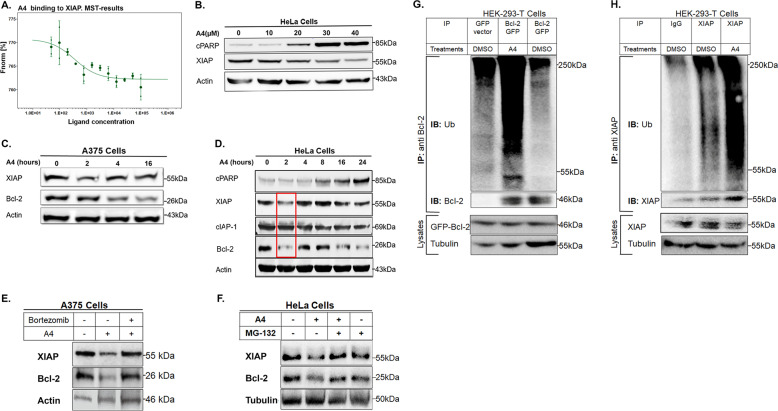
A4 binds directly to XIAP and promotes proteasome-mediated degradation of both XIAP and Bcl-2. **a** MST (microscale thermophoresis) analysis of A4 binding to fluorescently labelled recombinant XIAP revealed a direct binding with *K*_d_ of 348 ± 228 nM. A4 did not bind to cIAP1. **b** A4 induces a dose-dependent decrease of XIAP, which correlates with increased apoptosis. WB analysis shows a dose-dependent increase of the apoptotic marker cleaved PARP (cPARP), which correlated with a dose-dependent decrease in XIAP levels in HeLa cells treated with A4 for 24 h (*n* = 3). **c** WB analysis revealed a reduction of Bcl-2 and XIAP levels in A375 cells following treatment with A4 (*n* = 3). **d** Representative WB analysis for the expression levels of XIAP, Bcl-2 cIAP1 and cPARP in HeLa cells treated with A4 for 2 h (*n* = 3). **e** Representative WB analysis for the expression of XIAP and Bcl-2 upon treatment with A4 for 2 h in the absence or presence of the proteasome inhibitor Bortezomib in A375 cells (*n* = 3). **f** Representative WB analysis for the expression of XIAP and Bcl-2 upon treatment with A4 for 2 h in the absence or presence of the proteasome inhibitor MG-132 in HeLa cells (*n* = 3). **g**, **h** In vivo ubiquitylation assay of Bcl-2 and XIAP following treatment with A4. HEK-293-T cells were transiently transfected with Ub-HA (**g**, **h**) and GFP-Bcl-2, or GFP-vector as a control (**g**). Cells were pre-incubated with MG-132 for 6 h and A4 was added for the last 2 h. Bcl-2 or endogenous XIAP proteins were pulled down with anti-Bcl-2 (**g**) or anti-XIAP (**h**), respectively, followed by immunoblotting with an anti-ubiquitin antibody (*n* = 3).

The endogenous ARTS protein binds directly to XIAP and Bcl-2^28,34^. Similarly, using MST assays we found that A4 showed a relatively high binding affinity for recombinant XIAP (*K*_d_ = 348 nM), but no binding to cIAP1 (Fig. 4a, and data not shown, CreLux). To examine whether A4 can mimic the function of ARTS, we tested whether A4 can stimulate the degradation of both XIAP and Bcl-2, a feature that distinguishes ARTS from other known pro-apoptotic proteins. We tested some of our other top-ranked compounds for their ability to reduce endogenous levels of XIAP and Bcl-2 in several cell lines. A4 showed a pronounced effect by decreasing both these target proteins and was selected for further characterization (data of other compounds is not shown). A dose-dependent decrease in XIAP levels in A4-treated HeLa cells was associated with increased apoptosis (Fig. 4b). Reduction of XIAP and Bcl-2 levels was seen as soon as 2 h following treatment with A4 (Fig. 4c, d). This effect is specific as no reduction of cIAP1 was observed at this time point (Fig. 4d). Next, we found that addition of two different proteasome inhibitors (MG-132 and Bortezomib) to A4-treated cells restored levels of XIAP and Bcl-2 in A375 and HeLa cells, respectively (Fig. 4e, f). Finally, using in vivo ubiquitylation assays, we showed a substantial increase in accumulation of poly-ubiquitylated forms of Bcl-2 and XIAP in A4-treated HEK-293-T (Fig. 4g, h). This suggests that A4-induced reduction of XIAP and Bcl-2 protein levels occurs through ubiquitin-proteasome system (UPS)-mediated degradation. Since Bcl-2 acts by binding and neutralizing the pro-apoptotic function of Bax, and because A4 reduces Bcl-2 levels, we also examined if the levels of Bax affected A4-induced apoptosis. For this purpose, we treated Bax/Bax DKO MEFs with A4 and compared them with WT MEFs. We observed a significant reduction of caspase-9 activity and cPARP in the Bax/Bax DKO MEFs as compared to WT MEFs (Supplementary Fig. 2a). This suggests that A4-induced killing is affected by Bax. We conclude that A4 mimics the function of ARTS with respect to UPS-mediated degradation of XIAP and Bcl-2, the activation of caspase-3 and -9, and the induction of apoptosis.

### A4 kills specifically by targeting XIAP

**Fig. 5:**
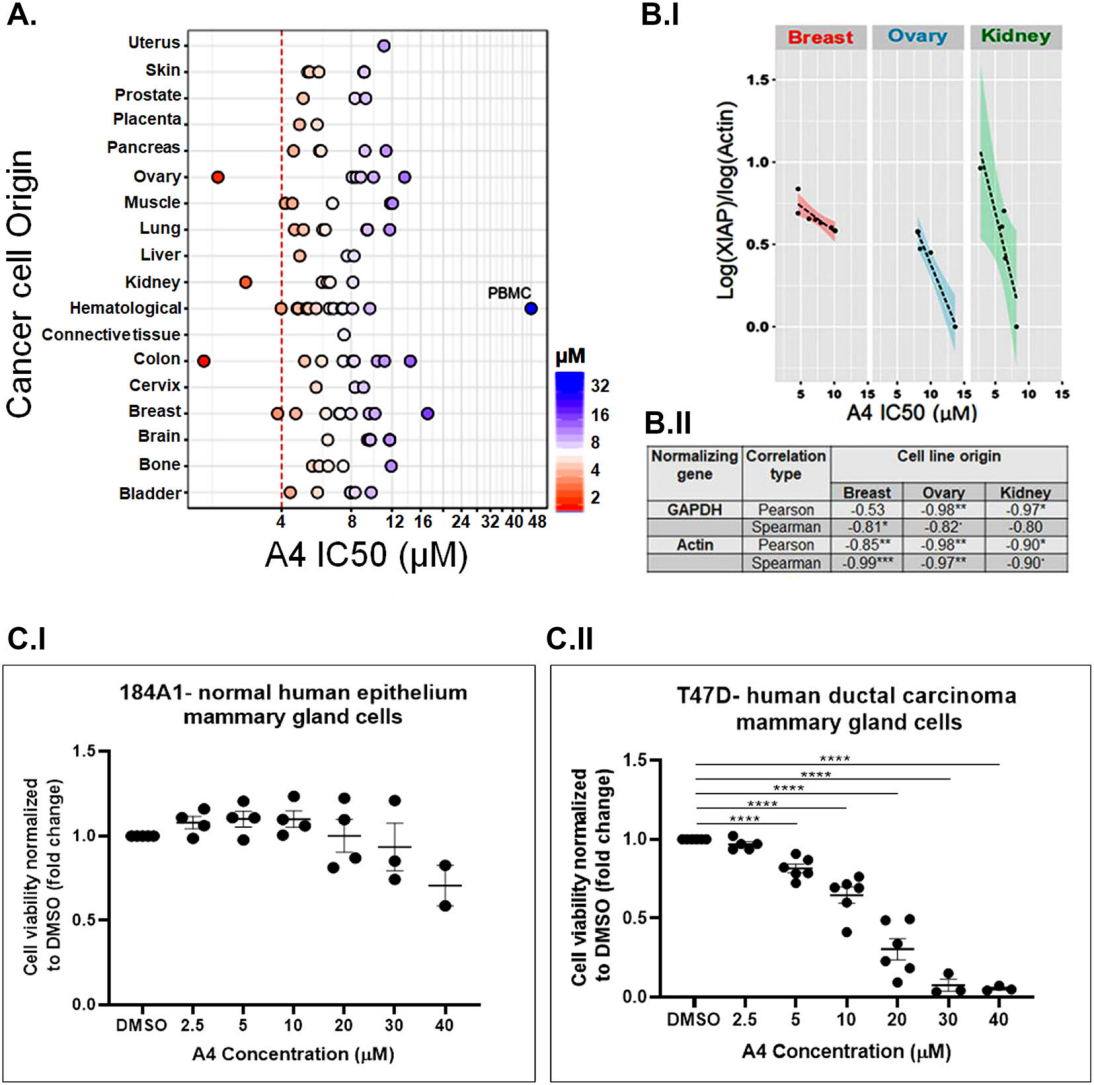
Differential cancer cell killing by A4. **a** IC50 values (μM) for a panel of 94 tumour cell lines (NCI-60 extended panel) treated with A4. Six concentrations of A4 were used to calculate the IC50 values for each cell line. Differential cell death effects of A4 on various cancer cell lines were observed. Normal PBMCs (peripheral blood mononuclear cells) were resistant to killing by A4. **b** (I) Scatterplots representing expression of XIAP normalized to actin levels in proteomes. Significant correlations between high levels of XIAP and sensitivity to killing by A4 were found in breast, ovary and kidney cancer cell lines. Dashed lines represent linear regression; shades represent 95% confidence area for the regression. (II) Following normalization of XIAP expression levels to either GAPDH or actin, Pearson and Spearman correlations were calculated and tested for significance. Significance codes: “*p* < 0.05”; **p < 0.01; ***p < 0.001. **c** (I, II) Cell viability was measured in 184A1 normal human mammary gland cells (*n* = 4) and T47D human ductal breast carcinoma cells (*n* = 6) upon treatment with different concentrations of A4 for 24 h. Dots represent an average of triplicate experiments. Bars; SEM, one-way ANOVA, ****p < 0.0001.

**Fig. 6:**
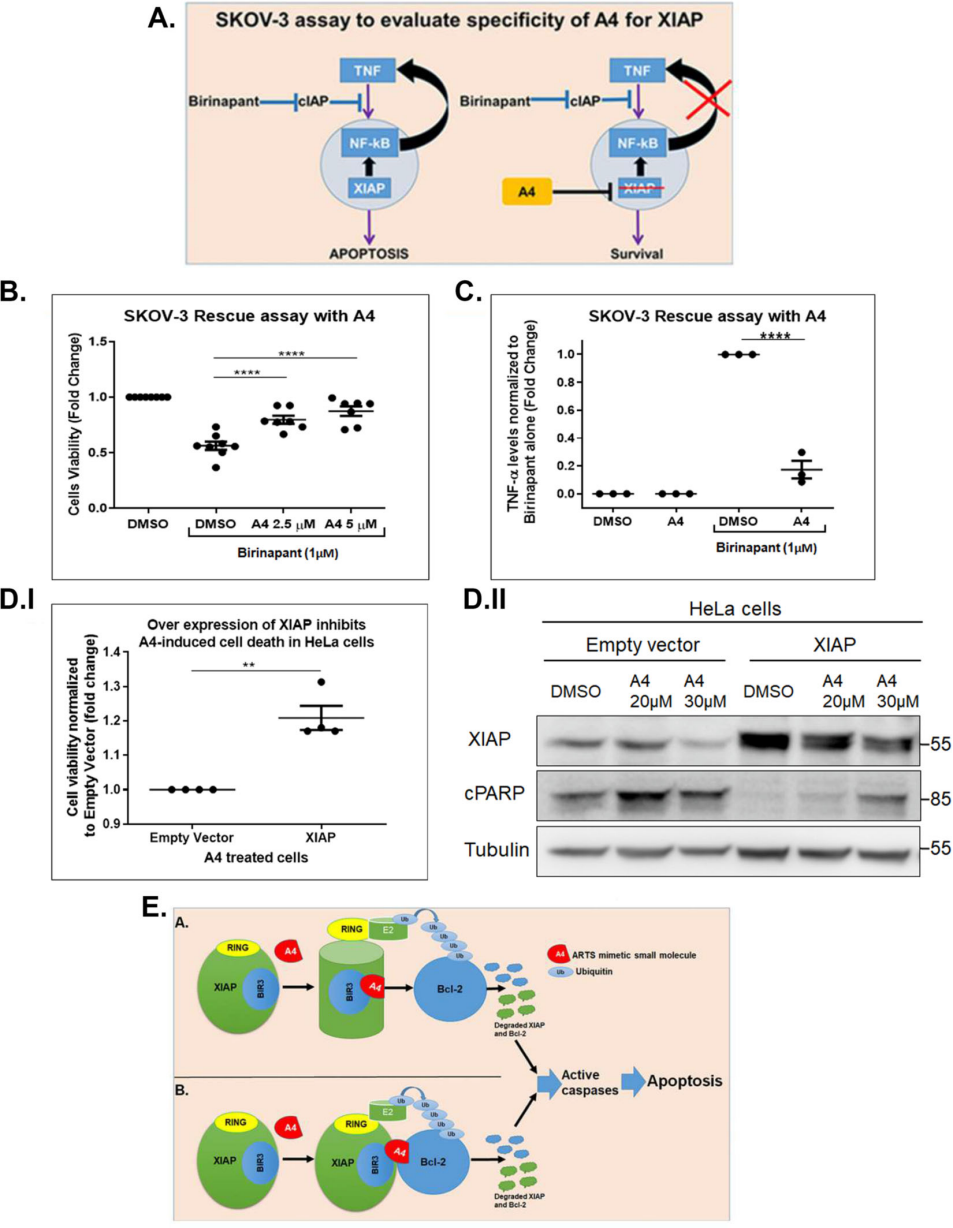
A4 specifically promotes degradation of XIAP. **a** Illustration of the SKOV-3 assay to evaluate specificity of A4 for XIAP antagonism. SKOV-3 cells are effectively killed by SMAC mimetics, such as Birinapant. Inhibition of cIAP1 by Birinapant leads to apoptosis through an autocrine feedback loop involving TNFα and NF-κB. XIAP function is required for Birinapant-induced killing in this system^49^. **b** Cell viability of SKOV-3 cells treated with Birinapant (1μM) in the absence or presence of A4 (*n* = 6). Dots represent an average of triplicate experiments. Bars; SEM, one-way ANOVA, ****p < 0.0001. **c** TNFα levels (pg/ml) measured in supernatants from SKOV-3 cells treated with DMSO, A4, Birinapant (1μM), and with the combination of Birinapant with A4 as determined by ELISA (*n* = 3). Dots represent an average of triplicate experiments. Bars; SEM, one-way ANOVA, ****p < 0.0001. **d** (I) Over-expression of XIAP inhibits A4-induced apoptosis. HeLa cells were transfected with either empty vector or XIAP and treated with A4 for 24h. Cell viability was determined using the Presto blue reagent. Cell viability measured in A4-treated cells transfected with XIAP was normalized to empty vector-transfected cells (fold change). Over-expression of XIAP led to an inhibition (1.2-fold change) of A4-induced cell death (*n* = 4). Dots represent an average of triplicates for each independent experiment. Bars; SEM, one-way ANOVA, **p < 0.01. (II) Representative WB analysis for the expression of XIAP and cPARP in HeLa cells and HeLa cells over-expressing XIAP upon treatment with A4 (*n* = 3). **e** Proposed model for the mechanism of A4-induced apoptosis. We consider two main models for the mechanism of A4 action (**a**). Upon binding to XIAPs, A4 induces an allosteric conformational change that results in the activation of XIAP E3 ligase activity, possibly by facilitating interaction of the RING motif with ubiquitin-conjugating (E2) enzymes. In this model, A4 would lead to a general increase in XIAP-mediated ubiquitylation, thereby explaining the increase in proteasomal degradation of both XIAP and Bcl-2. **b** In an alternative model, A4 binds directly to both XIAP and Bcl-2 with either low affinity or for a short time (“hit and run”). This recruits XIAP into close proximity with Bcl-2 and stimulates its ubiquitylation and proteasome-mediated degradation. In both model options, reduction of XIAP and Bcl-2 levels in cancer cells “addicted” to high levels of these proteins will enforce initiation of apoptosis. We suggest that A4 represents a novel class of ARTS-mimetic compounds acting by dual degradation of its target proteins.

To determine the specificity of A4 killing for various types of cancer, a panel of 94 cancer cell lines representing 18 different types of cancers were tested. The IC50 values for each cell line treated with A4 were determined (Oncolead Ltd). The cancers that were most sensitive to A4 killing were of haematological origin (8/12 different cell lines), melanoma (3/5 lines), pancreatic cancer and 4/7 lung cancers. Importantly, peripheral blood mononuclear cells (PBMCs), representing healthy normal cells, and 184A1, a normal human epithelium mammary gland cell, were not killed by A4 (Fig. 5a, Supplementary Table 1 and Fig. 5cI, respectively). To explore whether the levels of XIAP and Bcl-2 protein affect the sensitivity to A4, we analysed the available web proteomes and looked for a correlation. While data regarding XIAP expression levels were found in most proteomes (28 of 32 proteomes), Bcl-2 expression data were only detected in few (9 of 32 proteomes) (data not shown). A significant correlation was found between XIAP expression levels (ratio to actin, GAPDH) and the sensitivity to A4 (IC50, µM) in breast, ovary and kidney carcinomas (Fig. 5bI, II and Supplementary Tables 1 and 2). Cancer cell lines with high levels of XIAP were particularly sensitive to the killing effect of A4 (Fig. 5bI, II and Supplementary Tables 1 and 2). These results support the idea that A4 is a specific XIAP antagonist, and they suggest that A4 has the potential for treating cancers over-expressing XIAP. To further confirm that A4 functions as a specific antagonist for XIAP, we performed a SKOV-3 rescue assay^49^. SKOV-3 ovarian carcinoma cells are highly sensitive to SMs^49^. SMs act by antagonizing cIAPs, an event that leads to cell death when complemented by an XIAP-dependent increase in TNFα production^47,48,58^. In SKOV-3 cells, XIAP is required for TNFα production, which recruits cIAP into a complex that activates caspase-8 and apoptosis through the extrinsic pathway (Fig. 6a)^49^. Knockdown of XIAP in SKOV-3 cells bestowed resistance to SMs^49^. We utilized this assay to investigate the specificity of A4 for XIAP compared to cIAPs. Treatment with 1 μM of the SM Birinapant for 24 h killed ~50% of SKOV-3 cells. Importantly, addition of A4 to Birinapant-treated cells significantly blocked Birinapant-induced apoptosis (Fig. 6b). Moreover, Birinapant treatment resulted in secretion of TNFα into the media of SKOV-3 cells, and addition of A4 significantly reduced secreted TNFα (Fig. 6c). Next, we found that over-expression of XIAP abolished A4-induced cell death (Fig. 6dI, II). Collectively, these results indicate that A4 mimics the function of ARTS by inducing apoptosis through specifically antagonizing XIAP. Based on our results we propose two alternative models for the mechanism of A4 action. The first model proposes that upon binding to XIAP, A4 induces an allosteric conformational change that results in the activation of XIAP E3 ligase activity, possibly by facilitating interaction of the RING motif with ubiquitin-conjugating (E2) enzymes. In this model, A4 causes a general increase in XIAP-mediated ubiquitylation and degradation of both XIAP and Bcl-2. An alternative model is that A4, like ARTS, binds directly to both XIAP and Bcl-2 with either low affinity or for a short time (“hit and run”). This recruits XIAP into close proximity with Bcl-2 and stimulates ubiquitylation and proteasome-mediated degradation of the complex. In both models, reduction of XIAP and Bcl-2 levels in cancer cells “addicted” to high levels of these proteins initiate apoptosis (Fig. 6e).

## Discussion

We identified a small-molecule A4 that mimics at least some of the properties of the pro-apoptotic ARTS protein. A4 is a methyl 5-chloro-3-[[2-[4-(phenylmethyl) piperazin-1-yl]acetyl]amino]-1*H*-indole-2-carboxylat with a relatively simple chemical structure that conforms with Lipininski’s rule of five, making it well suited for possible future chemical modifications. Despite its small size, A4 recapitulates a number of specific biochemical and functional properties previously described for ARTS. First, like ARTS, A4 binds to XIAP-BIR3 but not cIAP1. Second, A4, like ARTS, can initiate the ubiquitylation and degradation of XIAP and Bcl-2, resulting in caspase activation and apoptosis (Figs. 4g, h, 3a–g)^13,34^. Third, A4 specifically antagonizes the function of XIAP, but not cIAP1, as demonstrated by inhibition of the apoptotic effect of A4 in XIAP-over-expressing cells (Fig. 6dI–II, 1c) and by its ability to suppress SM-induced cell death in the SKOV-3 assay (Fig. 6a–c). Collectively, these observations support the conclusion that A4 is a small-molecule ARTS mimetic. A4 was identified by showing highest docking affinity to the ARTS binding epitope in XIAP-BIR3, which is distinct from that of SMAC (Fig. 1b, d)^14^. This is in stark contrast to all currently published XIAP ligands that bind to the SMAC binding site of XIAP (Fig. 1a). These findings have important implications and suggest that the ARTS binding site in XIAP is “druggable”, and that A4 is a promising candidate for future development as a distinct anti-cancer drug.

Dual down-regulation of both major anti-apoptotic proteins, XIAP and Bcl-2, has been reported to cause enhanced apoptosis, increased sensitivity to chemotherapy and can overcome resistance of cancer cells^59–61^. To the best of our knowledge, A4 is the first compound shown to promote degradation of both these important anti-apoptotic proteins.

Significantly, cancer cells expressing high levels of XIAP were particularly sensitive to killing by A4, whereas no effect was observed on non-malignant PBMCs (Fig. 5a, b and Supplementary Table 1) and on 184A1 normal human epithelium mammary gland cells (Fig. 5cI). This suggests that A4 has selective activity towards cancer cells.

Bcl-2 is an important target for cancer therapy since it is highly expressed in a wide range of cancers, which renders them resistant to apoptotic stimuli and chemotherapeutic agents^18,21,23,62,63^. A series of high-affinity, small-molecule Bcl-2 inhibitors have been developed^64,65^, and the BH3-mimetic compound and Bcl-2-specific inhibitor Venetoclax (ABT-199) has been approved by the Food and Drug Administration for cancer treatment^66–70^. However, it remains an important goal to extend the utility of these agents to other types of cancer^71,72^. XIAP is another promising drug target for cancer therapy^38,45,46,73^. Silencing of XIAP was found to sensitize cells to chemotherapy and TRAIL receptor agonists and reduce resistance to chemotherapy^45^. In addition, XIAP can form heteromeric complexes with other IAPs and regulate cIAP stability^74,75^. Interestingly, targeting XIAP can bypass Bcl-2-mediated resistance to TRAIL and cooperate with TRAIL to suppress pancreatic cancer growth in vitro and in vivo^76^. Together, these observations point to a connection between the intrinsic and extrinsic apoptotic pathways, and specifically to the overlapping roles of XIAP and Bcl-2 for the regulation of cell death. Because our results suggest a dual function of A4 as an inhibitor of both XIAP and Bcl-2, it may be effective against a wide range of tumours over-expressing either one or both these proteins.

The vast majority of compounds that are currently developed as IAP antagonists (mainly SMs) act by binding and inhibiting XIAP rather than degrading it^38,73^. Similarly, BH3 mimetics, such as Venetoclax, act by binding and inhibiting Bcl-2^20,72^. Therefore, another attractive feature of A4 is that it stimulates the UPS-mediated degradation of both XIAP and Bcl-2 rather than binding and inhibiting its function (Fig. 4b–h). Target protein degradation has emerged as a promising therapeutic strategy, particularly in cancer, and proteolysis-targeting chimaeras (PROTACs) are currently developed^18,77,78^. Advantages include the potential to reduce systemic drug concentration and its possible accompanying cytotoxic side effects, and the ability to reduce the load of increased expression of target protein, which are often inhibitory proteins^78^. Since many types of cancers become “addicted” to high levels of Bcl-2 and/or XIAP, we propose that drugs that can promote their degradation would be of significant interest. In this context we suggest that A4 can have a considerable promise in this regard. Similar to PROTACS, A4 promotes UPS-mediated degradation of its substrates (Fig. 4g, h), and it may be possible to exploit this feature to develop drugs with substantially improved therapeutic abilities.

We consider two main models for the mechanism of A4 action: (A) upon binding to XIAP, A4 induces an allosteric conformational change that results in activation of XIAP E3 ligase activity, possibly by facilitating interaction of the RING motif with ubiquitin-conjugating (E2) enzymes. In this model, A4 causes a general increase in XIAP-directed ubiquitylation, thereby explaining the increase in proteasome-mediated degradation of both XIAP and Bcl-2. A similar mechanism involving a conformational change was shown for the action of SMs on cIAP1^79,80^. In order to explain A4-induced degradation of Bc-2, this model proposes that A4 stimulates the general E3 ligase activity of XIAP, perhaps by inducing a conformation that is more favourable for interaction with an E2 ubiquitin-conjugating enzyme (Fig. 6e). Moreover, this model assumes important kinetic differences in the poly-ubiquitylation and degradation of Bcl-2 versus XIAP: (A) Bcl-2 would be the preferred substrate during the initial phase, followed by self-conjugation of XIAP. (B) An alternative model is that A4 binds directly to both XIAP and Bcl-2, thereby recruiting them into close proximity to stimulate ubiquitylation and proteasome-mediated degradation (Fig. 6e). In either model, reduction of both XIAP and Bcl-2 in cancer cells “addicted” to high levels of these proteins stimulates apoptosis.

Overall, we described here the identification of an ARTS-mimetic compound, which represents a novel promising approach for development of cancer therapy. We suggest that A4 represents a novel class of ARTS-mimetic compounds acting by dual degradation of its target proteins and provides a promising starting point for the potential development of distinct anti-cancer drugs.

## Supplementary information

Supplementary Figure 1

Supplementary Figure 2

Supplementary Tables

Supplementary figure legends and Materials and methods
