# Supplementary figures and images for: A small-molecule ARTS mimetic promotes apoptosis through degradation of both XIAP and Bcl-2
Source: Cell Death Dis. 2020 Jun 25;11(6):483. doi: 10.1038/s41419-020-2670-2 (PMC7316745; doi:10.1038/s41419-020-2670-2)

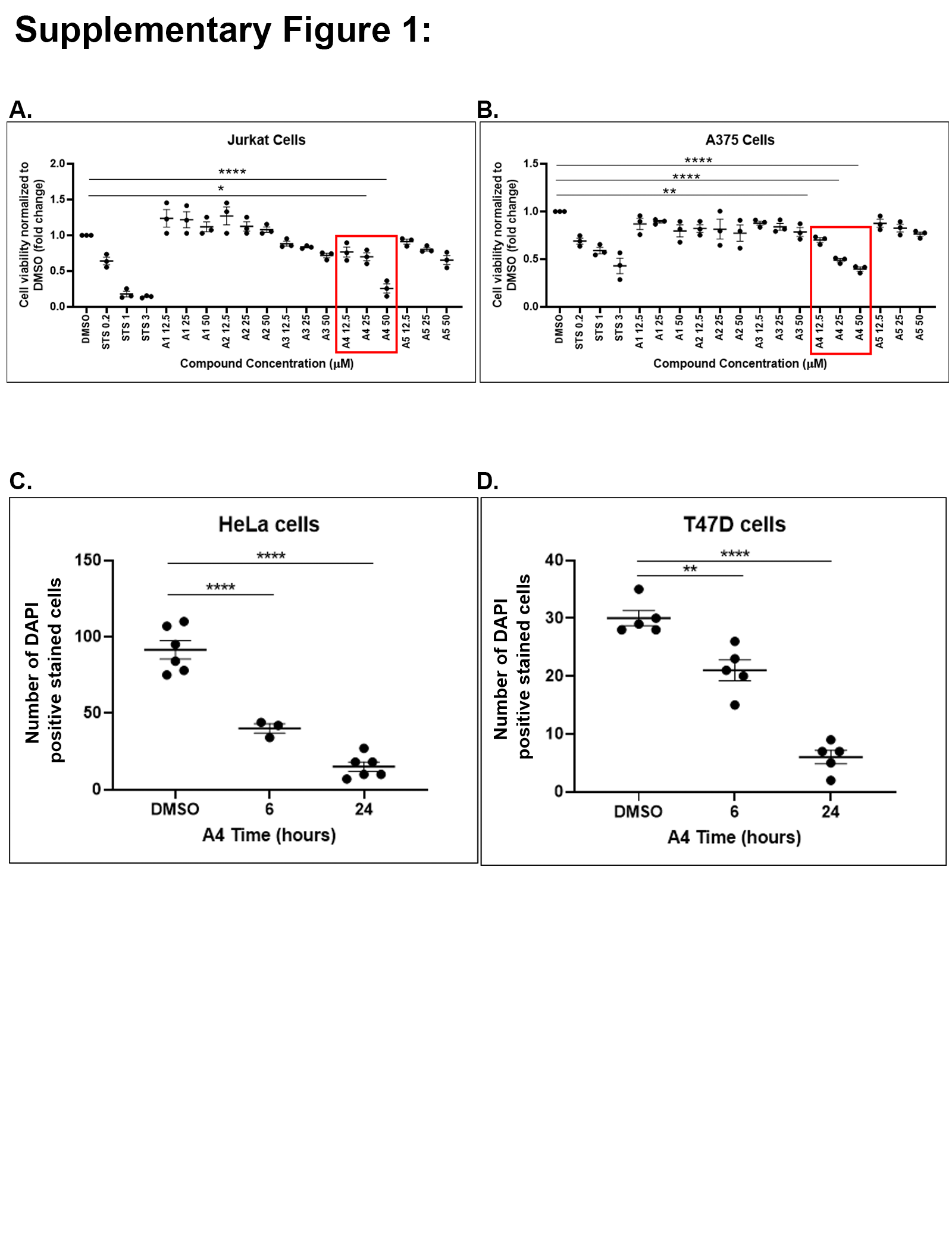

Supplement: Supplementary file 1 — Supplementary Figure 1 [file 41419_2020_2670_MOESM1_ESM.tif]

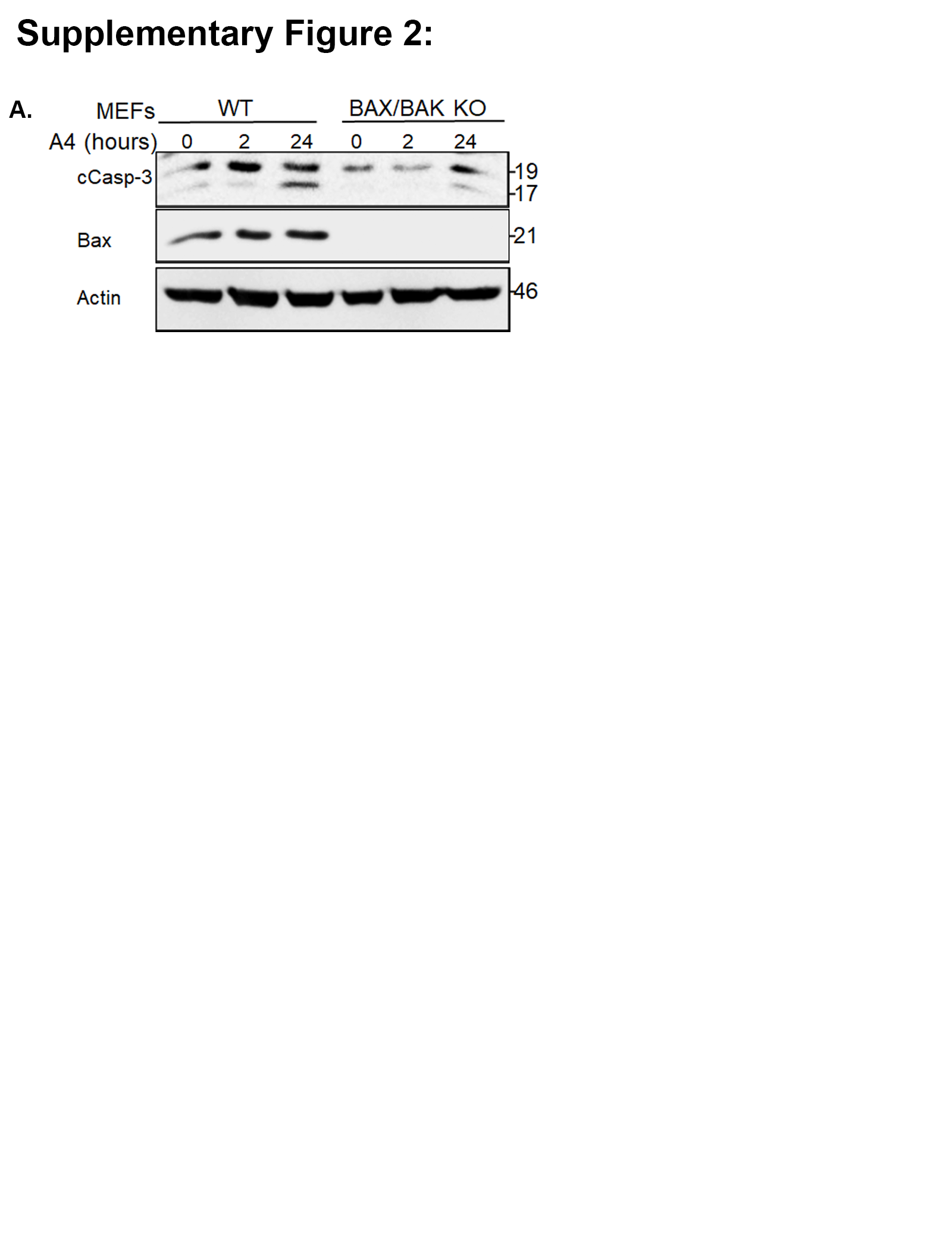

Supplement: Supplementary file 2 — Supplementary Figure 2 [file 41419_2020_2670_MOESM2_ESM.tif]
