# Supplementary material for: A small-molecule ARTS mimetic promotes apoptosis through degradation of both XIAP and Bcl-2
Source: Cell Death Dis. 2020 Jun 25;11(6):483. doi: 10.1038/s41419-020-2670-2 (PMC7316745; doi:10.1038/s41419-020-2670-2)
Supplement: Supplementary file 4 — Supplementary figure legends and Materials and methods [file 41419_2020_2670_MOESM4_ESM.docx]

**Supplementary figure 1. (A-B**) **A4 induces cell killing in a dose-dependent manner**. The highest-ranked docking compounds from our screen were evaluated for their cell killing ability in Jurkat and A375 cells using the PrestoBlue viability reagent. Dots represent an average of triplicates for each independent experiment, Bars; represents the mean. Error Bars; SEM, one-way ANOVA,, p<0.05*, p<0.01**, p<0.0001****
**C)** DAPI- positive HeLa cells were counted from immunofluorescence images shown in Fig.3A.I The average of total cell counts from 5 images were taken for each treatment and time point. **D)** Counts of T47D cells were done as described in above in C (n=3) Dots represent an average of triplicates for each independent experiment, Bars; represents the mean. Error Bars; SEM, one-way ANOVA,, p<0.05*, p<0.01**, p<0.0001****). A significant decrease in the number of viable cells is observed after 6 and 24 hours of treatment with A4.

**Supplementary figure 2.A)** Representative WB of cleaved caspase-3 to evaluate induction of apoptosis by A4. Levels of cleaved caspase-3 increased after 2 and 24 hours of A4 treatment in WT MEFs, but much less in BAX/BAK KO cells (n=3). This suggests that BAX/BAK contribute to A4-induced apoptosis through the mitochondrial apoptotic pathway.

**Supplementary Table 1)** A4 IC50 values, XIAP, Bcl-2, actin, GAPDH expression values in proteomes from 32 representative cancer cell lines used in the cancer cell line panel screen (shown in Figs.5A, B).

**Supplementary Table 2)** Number and names of cell line proteomes shown in Fig. 5BI, II.

**Supplementary Materials and methods**

**Site directed mutagenesis of XIAP expression construct**

XIAP was mutated in its ARTS binding pocket. Specifically, using a GFP-WT-XIAP construct (pcDNA3-GFP-XIAP), we mutated the following amino acids: S278A and N280A using two step site directed mutagenesis. In brief, two separate PCR reactions were done with the following set of primers first set F: 5`-GGATATACGCAGTTGCCAAGGAGCAGC-3`, R: 5`-TAAACGGGCCCTCTAGATTAAGACATAAAAAT-3`and second set F: 5`-GCTGTACAAGCTCGAGACTTTTAACAGTTT-3`, R: 5`-CTTGCAAGCTGCGCCGCGTTAACTGAG-3`. Fragments generated from both PCR reactions were mixed together and used as a DNA template for the third PCR reaction. This PCR was done using the following primers : F. 5`-GCTGTACAAGCTCGAGACTTTTAACAGTTT-3` and R: 5`-TAAACGGGCCCTCTAGATTAAGACATAAAAAT-3`. The generated fragment was cut with XhoI and XbaI restriction enzymes and inserted into pcDNA3-GFP vector cut with the same restriction enzymes. The DNA sequence of the GFP-XIAP S278A/N280A mutant was approved and its expression was confirmed by Western blot. We refer to this mutant form of XIAP as GFP-Double-mutant-S278A-N280A-XIAP (or GFP-DM-XIAP). As a control we used the GFP-WT-XIAP and the GFP-empty-vector. GFP tag is attached to XIAP to the N-terminus.

**Computational screen**.

300,000 commercially available molecules were selected from a set of ~3 million and were screened using LeadIT and SeeSAR software suits from BioSolveIT. This computational screen identified compounds with predicted binding affinities in the micromolar to nanomolar range, as assessed by the HYDE scoring function ^1^. The 100 top-ranked molecules exhibiting best docking scores were determined. The ARTS unique binding site in XIAP-BIR3 was extrapolated by analysing XIAP- SMAC crystal structures from the PDB and our data, described in Bornstein et al. ^2^.All XIAP structures containing the ARTS binding site (amino acids residues 272-292 of XIAP: FGTWIYSVNKEQLARAGFYAL) were downloaded, but only regions with x-ray crystal structures with a resolution better than 2.7 Angstroms were further investigated. Analysis of the putative ARTS-XIAP binding site was performed using the DoGsitescorer ^3^. Relevant pockets were only considered to be those involving the above amino acids, but not pockets covering the SMAC binding site. Overlay of the two binding sites with the biggest volumes (3HL5 and 3CLX) indicated that these sites share a very similar overall topology, except for one amino acid (Phe270), which in 3CLX adopts a more “packed down” conformation. On the basis that the 3HL5 (chain A) structure is more highly resolved and adopts a Phe270 side chain conformation, which generates a more defined “pocket”, this structure (3HL5) was chosen as the basis for defining the binding site. To ensure the reliability of that binding site, the B-factors were checked. There were approximately 10 B-factors for each atom in this site, which indicated that all atoms were positioned with high confidence.

The binding site for the docking studies was built to include all residues in the close neighbourhood of the identified binding pocket, starting from chain A of 3HL5. Water molecule HOH-25-A was retained; all others were removed. A single SMARTS spatial constraint was added (a single heavy atom [*] must be within the specified sphere within the binding pocket) to ensure the placement of the molecules by the docking algorithm in the desired area. The basis for the small molecule input set was the eMolecules database December 2013 release, containing 5,488,934 structures in SDF format. Feature-Trees were generated using FTrees Version 2.4. Molecular descriptors (canonical SMILES, SLogP and Exact MW) were generated using RD Kit as provided under https://tech.knime.org/community/rdkit. Molecule processing was accomplished using the KNIME workflow package (KNIME GmbH). Molecules with missing descriptors were removed. Molecules with SLogP greater than 4.1 were then removed, followed by those with molecular weight less than 125 amu and greater than 450 amu. Duplicates were removed by matching canonical SMILES strings and converted to mol2 format for docking and scoring. This processing generated a compound set of 3,529,046 unique molecules.

60,000 molecules were randomly selected from the entire filtered eMolecules set. These were then docked (20 poses) and scored with FlexX ^4^, and re-scored with HYDE ^1^. Poorly fitting poses were removed by filtering by intermolecular Lennard-Jones potential (removed all >1000). The top 1000 scoring molecules were selected and used as queries in an FTrees ^5^ similarity search of the entire eMolecules filtered set. The 50 most similar molecules and 10 diverse 0.8 similarity-score molecules to each of the 1000 queries (i.e. 60,000 new molecules) were docked and re-scored as described as before. Poorly fitting poses were again removed by filtering by intermolecular Lennard-Jones potential (removed all >1000). This process was then repeated four times. The final set of molecules was extracted from these results after filtering by intermolecular Lennard-Jones potential (<1000) and ligand efficiency (>1.5). The top 1000 hits from the entire screening exercise were filtered by HYDE score (better than -36 kJ/mol) and then by intermolecular Lennard-Jones potential (< 500). This reduced the size of the dataset to 348 molecules. As a preliminary counter-screen, these 348 hits were re-docked (35 poses, FlexX) and re-scored (HYDE) in XIAP PDB structure 1G73 using the same amino acids used to define the binding site in 3HL5 and with a water molecule manually added into the same position as water HOH-25-A from 3HL5 (waters are missing from structure 1G73). Only molecules scoring better than -26.5 kJ/mol were retained (120 molecules). The predicted binding poses were then manually analysed to confirm acceptable conformations and binding mode and 100 of them were selected for testing.

**MST binding assays**

MST binding assays were performed by CreLux, a WuXi AppTech company in Germany, using recombinant ARTS, XIAP, Bcl-2 and cIAP1 proteins. Specifically, for performing experiments with untagged XIAP, a fluorescent label (NT650) was covalently attached to the protein (Maleimide coupling). The labelling was performed in a buffer containing 50 mM Hepes pH 7.0, 150 mM NaCl and 0.005% Tween 20. A detailed description is in the supplementary materials and methods.

Binding experiments were performed on the Monolith NT Automated instrument using the pico RED detector with an XIAP untagged concentration of 10 nM. In all labelled MST experiments, the concentration of the fluorescent molecule was kept constant while the concentration of the non-fluorescent molecule (= ligand) was varied with 24 samples prepared at different concentrations in duplicates. Compounds were screened in a twenty-four point twofold serial dilution beginning at 250 μM compound concentration down to 0.03 nM, with a DMSO concentration of 5%. The assay was performed in buffer containing 50 mM Tris pH 7.5, 150 mM NaCl, 0.005% Tween 20 and 0.1% PEG 8000. After 10 min incubation the samples were loaded into MonolithTM NT automated MST premium coated 24-capillary chips. The MST experiment was performed with 10% LED Power and medium MST Power. A Kd was determined with the MO. Affinity Analysis Software v2.2.4 from Nanotemper.

**References**

1. Schneider, N., Lange, G., Hindle, S., Klein, R. & Rarey, M. A consistent description of HYdrogen bond and DEhydration energies in protein-ligand complexes: methods behind the HYDE scoring function. *J Comput Aided Mol Des* **27**, 15-29 (2013).

2. Bornstein, B. *et al.* ARTS binds to a distinct domain in XIAP-BIR3 and promotes apoptosis by a mechanism that is different from other IAP-antagonists. *Apoptosis* **16**, 869-881 (2011).

3. Volkamer, A., Kuhn, D., Rippmann, F. & Rarey, M. DoGSiteScorer: a web server for automatic binding site prediction, analysis and druggability assessment. *Bioinformatics* **28**, 2074-2075 (2012).

4. Holavanahali, R. *et al.* Directly modulated diode laser frequency doubled in a KTP waveguide as an excitation source for CO2 and O2 phase fluorometric sensors. *J Biomed Opt* **1**, 124-130 (1996).

5. Rarey, M. & Dixon, J.S. Feature trees: a new molecular similarity measure based on tree matching. *J Comput Aided Mol Des* **12**, 471-490 (1998).
